# Finite element modeling of human Achilles tendon: impact of subtendon modeling and fiber direction

**DOI:** 10.1007/s10237-026-02132-z

**Published:** 2026-09-07

**Authors:** Chiara Garavelli, Pernilla Eliasson, Hanna Isaksson

**Affiliations:** 1 Department of Biomedical Engineering, Lund University, Box 118, 221 00 Lund, Sweden; 2 Department of Orthopaedics, Institute of Clinical Sciences, Sahlgrenska Academy, University of Gothenburg, Gothenburg, Sweden; 3 Department of Orthopaedics, Sahlgrenska University Hospital, Gothenburg, Sweden

**Keywords:** Strain distribution, Twist, Magnetic resonance imaging, In-silico model, Poro-viscoelasticity

## Abstract

The Achilles tendon is the thickest tendon in the human body, and it is composed of three subtendons connected to the calf muscles (soleus, medial and lateral gastrocnemius). This study aimed to evaluate the importance of explicitly modeling subtendons and collagen fiber orientation on the predicted strain distribution within tendon tissue during mechanical loading. Magnetic resonance images from seven subjects were used to create subject-specific finite element models. The segmented tendons were modeled in two ways: the entire tendon as one structure, then dividing it into the three subtendons. A fiber-reinforced poro-visco-hyperelastic material model was used to describe the mechanical response. The collagen fibers were first oriented in the proximal–distal direction, then twisted following the subtendon orientation. Principal strains were analyzed, assessing the impact of subject-specific geometry on strain magnitudes, localization and distributions. Average strains were similar across subjects (coefficient of variation = 18%), but location and magnitude of peak strains were subject-specific. While models including the subtendons predicted similar average strain values to those without, local heterogeneity increased substantially in the subtendon model (Full-Width at Half-Maximum increased from 0.03 to 0.08). Altering the collagen fiber orientation shifted the localization of higher strains (Full-Width at Half-Maximum = 0.11). In conclusion, both subtendons and realistic fiber orientation should be considered when modeling the mechanical behavior in the Achilles tendon as it strongly affects the strain distribution within the tendon. Accurate prediction of strain localization and distribution is important for further studies of tendon mechanobiology and for designing more effective rehabilitation protocols.

**Supplementary Information:**

The online version contains supplementary material available at https://doi.org/10.1007/s10237-026-02132-z.

## Introduction

The Achilles Tendon (AT) is the largest human tendon, bearing loads of 2.5–4.0 × body weight during walking, and up to 4.0–7.5 × body weight during running (Demangeot et al. 2023). Consequently, the AT is also the tendon most prone to injury (Vila Pouca et al. 2021). The incidence of AT ruptures is around 90 per 100,000 person-years worldwide (Bergamin et al. 2023), and it peaks around the ages of 20–50 years old (Järvinen et al. 2005). Rehabilitation after a tendon injury is long and often ineffective, partly as the protocols are often standardized and dependent on the specific physiotherapist for patient-specific adaptations. Consequently, many patients struggle to return to their pre-injury lifestyle (Johns et al. 2021).

Computational modeling has the potential to support clinical decision-making, as shown for other musculoskeletal conditions, including bone fracture risk prediction related to osteoporosis (Grassi et al. 2023) and cartilage degeneration prediction during osteoarthritis (Orozco et al. 2024). Thus, there is a large potential for implementing similar in-silico tools built directly from patient diagnostic images in the treatment of tendinopathies. Indeed, finite element (FE) modeling of the human AT has been exploited to predict the magnitude and the location of the peak strain during common rehabilitation exercises (Handsfield et al. 2020; Funaro et al. 2022), although a thorough validation is still required. Moreover, it has been shown that AT stresses strongly depend on subject-specific tendon geometry (Shim et al. 2014; Hansen et al. 2017).

However, some aspects regarding the implementation of these models remain to be addressed. The first point is linked to the Achilles tendon anatomy. The proximal aspect of the Achilles tendon connects to three distinct muscles: the medial and lateral gastrocnemius (MG and LG) and the soleus (SOL), often called the tripartite structure. This results in different tensile forces acting on the three segments of the tendon. In addition, the relative twist of the three subtendons has been found to vary between subjects in an ex vivo study of 53 cadavers (Pękala et al. 2017). Still, three main types of twisting were identified, ranging from moderate (type I, the most common, 48% occurrence) to extreme (type III, the least common, 6% occurrence) (Pękala et al. 2017). Recent studies implemented the tripartite structure of the Achilles tendon in FE models (Handsfield et al. 2020; Knaus and Blemker 2021; Yin et al. 2021; Funaro et al. 2022), and analyzed how strain peaks in the tendon change when different twist types are assumed (Handsfield et al. 2020). Some studies have instead modeled only the separation between the soleus and the gastrocnemius (Diniz et al. 2023, 2024), while others have considered the AT as a single structure (Hansen et al. 2017; Shim et al. 2018). While the tripartite structure of the AT and anatomic variation are recognized, it remains unclear if and how these structural features affect the magnitude and distribution of tensile strain.

Another important point for computational modeling is the choice of the material model. Tendons are mainly composed of water (55–70%) with the majority of their dry weight (60–85%) consisting of collagen type I arranged in a strong hierarchical fashion inside fibers and fascicles (Thorpe and Screen 2016), making them able to bear high loads. Unloaded collagen fibrils are crimped, and they start to straighten when tension is applied. This characteristic results in a nonlinear stress–strain response, featuring an initial toe region (~ 0–3% strain), followed by a linear region up to approximately 8–10%. Beyond this point, individual fibers begin to fail, resulting in a decrease in stiffness until macroscopic failure occurs (Wang et al. 2018). Furthermore, tendons are viscoelastic, as evidenced by various ex vivo experimental studies that loading the tendon under stress-relaxation (Ekiert et al. 2021), creep (Wren et al. 2003) and cyclic loading (Pierantoni et al. 2023). However, FE models of the human AT present in the literature have used either a hyperelastic or a viscoelastic material formulation (Thompson et al. 2017). Thus, the porous nature of the tendon and the effect of the fluid during tendon loading have largely not been accounted for. Nevertheless, the importance of including the fluid component in the tissue has been emphasized (Khayyeri et al. 2016), particularly regarding how the fluid flow contributes to tendon viscoelasticity (Yin and Elliott 2004). Additionally, the fact that the solid tendon matrix is saturated with water means that when tendons are loaded in tension the fluid moves through the porous matrix. This fluid movement leads to time-dependent local redistribution of the deformation. Consequently, a biphasic material formulation becomes important when the time dependency of the applied load plays a significant role.

Finally, the orientation and distribution of collagen fibers are highly important (Janssen et al. 2025). Currently, this information cannot be obtained from clinical imaging, as it requires micrometer-scale imaging technologies (Svensson et al. 2017; Zhou et al. 2018; Pierantoni et al. 2021, 2023). Different approaches have been adopted to represent this characteristic; in some cases, the fibers are modeled assuming proximal–distal alignment (Notermans et al. 2019), and in another case, heat flux simulations have been performed to address the fibers (Diniz et al. 2023). Nevertheless, the impact of collagen fiber orientation and distribution on the final deformation state of the tendon remains uncertain.

Consequently, the aim of this work was to present a pipeline for generating computational models of the human AT from clinical magnetic resonance images. It also aims to quantify the impact of explicitly modeling the subtendons on the strain distribution in the AT, as well as assessing the impact of altering the orientation of the collagen fibers. Accurate prediction of tendon strain patterns is essential for improving individualized rehabilitation, as it enables identification of regions at higher risk of overload or reinjury.

## Material and methods

### Patient data

Seven subjects (6 men and 1 woman) aged 31–64 years were scanned at the ankle level with magnetic resonance imaging (MRI) using a 3 Tesla scanner (Philips, MR systems Ingenia, version 5.9, 3.0 T). The patients were scanned with their foot in 90° flexion (anatomical neutral) within a body coil. A 3D T1-weighted spoiled gradient-echo sequence of the calf was used (TR/TE = 4.76/2 ms, flip angle 10°). The reconstructed voxel size was 0.98 × 0.98 × 3 mm^3^ (matrix 512 × 512, FOV 500 mm). The scan covered approximately 60 cm, measured from the calcaneal tuberosity, and included the distal thigh, the entire lower leg and the ankle joint with the calcaneus. The study received approval from the Swedish Ethical Review Authority (2019-5547, with an amendment 2024-04118-02). All patients had sustained an Achilles tendon rupture more than two years before undergoing MRI, but for this analysis, only images from the uninjured contralateral side, assumed to be clinically healthy after evaluation performed by a professional with expertise in musculoskeletal imaging, were used. Participant characteristics are summarized in Table 1.

**Table 1:** Morphometric information of the participants in the study, including height and weight for each patient, as well as the length of free tendon (*L*_0_) measured between the calcaneal bone and the inferior margin of the soleus muscle and tendon cross-sectional area (CSA)

Patient ID	Height (cm)	Weight (kg)	*L*_0_ (mm)	CSA (mm^2^)
#1	180	96	72.0	230
#2	180	88	53.5	190
#3	170	87	57.5	245
#4	178	65	51.0	179
#5	187	87	34.0	165
#6	172	78	40.2	288
#7	179	86	46.5	264

### Computational modeling

The work was divided into two parts. First, the FE models of the AT were generated considering the whole AT structure as one homogenous entity. Secondly, the modeling pipeline was modified to include an additional step that divided the AT volume into the three subtendons. All the generated models were simulated using the following described material properties and boundary conditions.

#### Volume generation

**Fig. 1:**
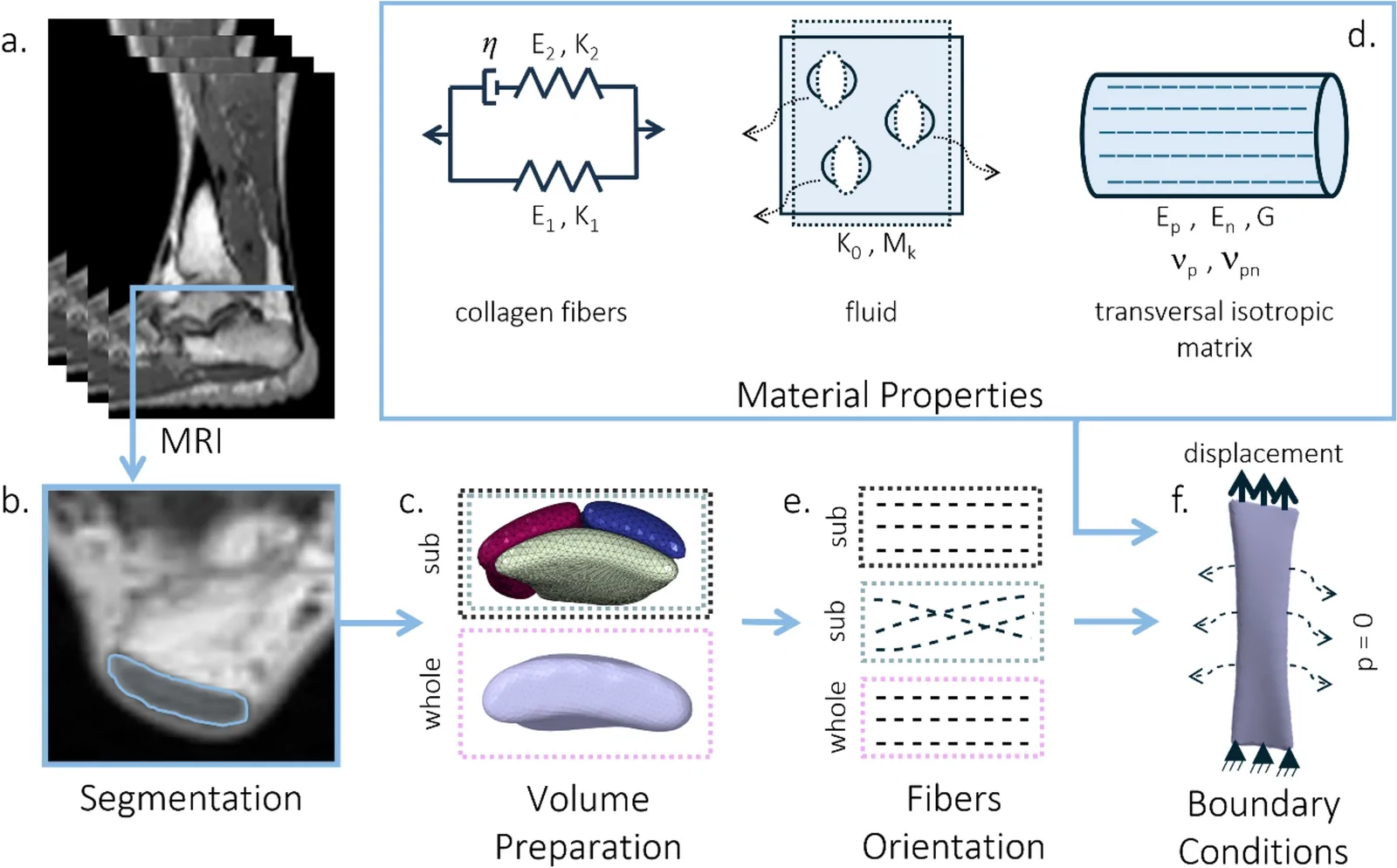
Study workflow. **a** The geometry of the Achilles tendon was extracted from the magnetic resonance imaging (MRI) of the participants. **b** The segmented surface was converted into a three-dimensional volume. **c** Two separate models were generated; a “whole” model where the tendon was considered as a single structure, and a “sub” model where the three subtendons were modelled explicitly. **d** A fiber reinforced poro-visco-hyper-elastic material model was used to describe the mechanical response, **e** with fiber oriented in either straight or twisted fashion. **f** The simulations were solved by applying displacement-controlled load to the top and allowing free fluid flow at the external surfaces

For each participant, the FE model was developed from the MRI scans (Fig. 1a). The free tendon, i.e., the tendon portion between the calcaneus insertion and the inferior margin of the soleus muscle, was manually segmented (3DSlicer, 5.6.2, Table 1) (Fig. 1b) and subsequently meshed with four-node linear tetrahedral pore pressure elements with an maximum edge length of 0.6 mm (Hypermesh 2021), resulting in around 65,000 elements and 20,000 nodes.

Starting from the segmented volumes described above, the whole tendon was further divided into the three subtendons comprising the AT (SpaceClaim, 2024R1). The subdivision was performed following the approach described by Knaus and Blemker (2021), in which the external tendon geometry was personalized, while the internal subtendon structure was based on a predefined template (Fig. S2). Specifically, at the proximal end of the free Achilles tendon (i.e., the soleus muscle–tendon junction), the cross-sectional area was partitioned into three regions corresponding to the muscle origins. Approximately 50% of the area was assigned to the soleus subtendon, and the remaining area was equally divided between the medial and lateral gastrocnemius subtendons (25% each), as described by Handsfield et al. (2020). It is important to note that this idealization was done as it was not possible to distinguish the internal separation between subtendons from standard MRI. The division lines were extruded through all the free tendon length until the calcaneus insertion, applying a proximal–distal torsion of 45 degrees based on the average twist found in literature (Łazarz et al. 2024) (Fig. 1c). The subtendon models included around 200,000 elements and 40,000 nodes.

#### Material model

A constitutive model describing the AT as a transversely isotropic fiber-reinforced poro-visco-hyperelastic material was used to describe the mechanical response. This advanced material model has previously been validated on rat Achilles tendons (Khayyeri et al. 2016; Notermans et al. 2019) but has not been used to model human tendons. Briefly, this material model integrates three main components: a non-fibrillar matrix of proteoglycans, the collagen fibers that are assumed to be load-carrying in tension-only and the fluid part, given the fact the matrix is considered porous and fully saturated with water (Fig. 1e). The transversely isotropic contribution of the non-fibrillar matrix is described by five parameters (the two elastic moduli *E*_n_ and *E*_p_—respectively, in the fiber direction and in the traversal direction—the Poisson’s ratio *ν*_pn_ and *ν*_p_, and the shear modulus G). The viscoelastic fiber response is simulated using a standard linear solid (SLS) model, where the springs are described with an exponential stress–strain relationship (σ = *E* (*e*^*kε*^—1)). This implementation of the SLS model requires five parameters, i.e., the stiffness parameters for the exponential behavior of the springs *E*_1_, *k*_1_, *E*_2_, *k*_2_, and the damping constant for the dashpot *η*. The tendon permeability is exponentially dependent on the change in void ratio upon deformation, described by initial permeability *k*_0_ and the exponential constant *M*_*k*_. More details on the material model are available in Supplementary Material S1.

Given that approximately 95% of the tendon’s mechanical response is attributed to the collagenous components (Khayyeri et al. 2016), the five parameters of the SLS model were optimized, while the other parameters were set to fixed values based on previous studies (Khayyeri et al. 2016; Notermans et al. 2019). The values assigned to the matrix parameters were *E*_n_ = 3.27 MPa, *E*_p_ = 6.80 MPa, *ν*_pn_ = 0.12, *ν*_p_ = 0.37 and *G* = 2.09 MPa, while *M*_k_ and *k*_0_ were set to 4.24×10^−1^ and 1.44×10^−9^ mm/s, respectively. The collagenous parameters were optimized by fitting simulated stresses (Matlab, R2022a) to experimental data extracted from the study by Ekiert et al. (2021). To the best of the authors’ knowledge, that study is the only experimental study on human AT with a strain range relevant for daily loading and a duration able to assess the viscoelastic behavior. Fifty human AT (aged 20–86 years) were subjected to three subsequent steps of traction: 3%, 6% and 9% strain, each followed by a relaxation period of 30 s. All loading steps were performed using 1% s^−1^ strain rate. To fit the material parameters, a procedure presented by our team for identifying the material parameters of cartilage tissue was used (Jönsson et al. 2024). Briefly, equal weights were assigned to eighteen points manually selected on the peak force region (three points), the rapid relaxation region (three points), and the equilibrium force region (three points) for each of the last two load steps. The root mean squared error (RMSE) between experimental and simulated stress was minimized using an algorithm based on the Method of Moving Asymptotes (Svanberg 1987) to solve the optimization problem. The fitting procedure was performed on one whole tendon model. The obtained parameters were subsequently used for all other simulated models.

For the subtendon models, two alternative primary fiber orientations were compared: A) the primary fiber orientation was maintained straight as in the whole tendon model approach, and B) the primary fiber orientation was adjusted to follow the twisting of each subtendon along the AT length (Fig. 1d). Specifically in the latter approach, the initial direction of the collagen fibers was defined elementwise as follows: firstly, the tendon length was divided into approximately 2-mm subsections, and the centroids of each subsection were computed. Fiber direction was then defined by connecting the centroids within each of the subtendons. All the elements within a subsection were assigned to the fiber direction computed for that subsection (Fig. S3). In both approach A and B, each element of the model had one identified fiber direction.

#### Boundary conditions

The boundary conditions of the model were set to reproduce the experimental loading protocol (Ekiert et al. 2021). Consequently, a displacement-controlled load was applied to the top extremity, while the bottom was fixed and free fluid flow was allowed through all the external surfaces, except for the boundary regions (Abaqus, 2021, Dassault Systèmes) (Fig. 1f). Three subsequent steps of traction were applied (3%, 6% and 9% strain), each one of them followed by keeping the load constant for 30 s.

For the subtendon models, frictionless contact in the tangential direction and hard contact with no separation after the first contact in the normal direction were assumed between structures.

### Outcome analysis

For each model, maximum principal strains were extracted at all the nodes. The location of the peak strains and the strain distribution in the proximal–distal direction (from the calcaneus to the muscle–tendon junction) and in the mediolateral direction (from the internal to the external side) were analyzed to assess the impact of subject-specific tendon geometry. Here, to enable consistent comparison between tendons with different dimensions, the node locations were normalized to the free tendon length (for the proximal–distal coordinate, where 0% corresponds to the calcaneal insertion and 100% corresponds to the soleus muscle origin) and width (for the mediolateral coordinate, where 0% corresponds to the location of the most medial node and 100% to the most lateral node). The tendons were then divided into twenty equally sized segments, within which the average, maximum and standard deviations were computed.

The strain distributions were compared between the whole tendon and the subtendon models, with and without twisting fiber orientation. Full-Width at Half-Maximum (FWHM) was computed to quantify the effects induced by the inclusion of a) explicit subtendon modeling and b) twist of the main fiber direction on the maximum principal strains magnitude predicted by the models after the last relaxation step (9% strain). FWHM analysis is explained in Supplementary material S2.

Moreover, the distributions of the strains at the nodes of the three subtendons considered separately were compared to assess the location of the strains. The purpose of this sub-analysis was to assess whether one specific subtendon was experiencing higher strain concentration compared to the others.

To assess the effects of tendon subject-specific geometry (7 subjects) and type of tendon model (whole vs. subtendon), an Aligned Rank Transform ANOVA, a nonparametric method suitable for factorial designs with non-normal data distributions (Wobbrock et al. 2011), was used on the average as well as on the maximum nodal strain values at the end of the third load step (9% peak strain). The significance level was set at *α* = 0.05.

Lastly, the effect of subtendon modeling on the fluid behavior was assessed by comparing the outflow at the highest load applied, before and after the relaxation period.

## Results

**Fig. 2:**
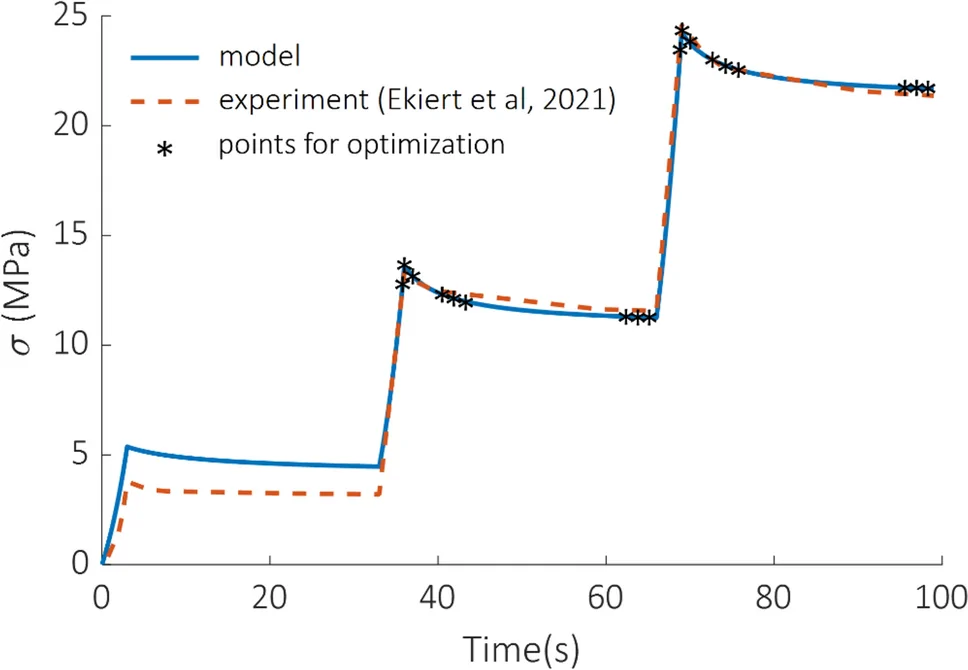
Model calibration. The poro-visco-hyperelastic material model (blue) fitted to experimental data (orange) on human Achilles tendons obtained from the literature (Ekiert et al. 2021). The points where the optimization error was computed are reported with black stars

**Fig. 3:**
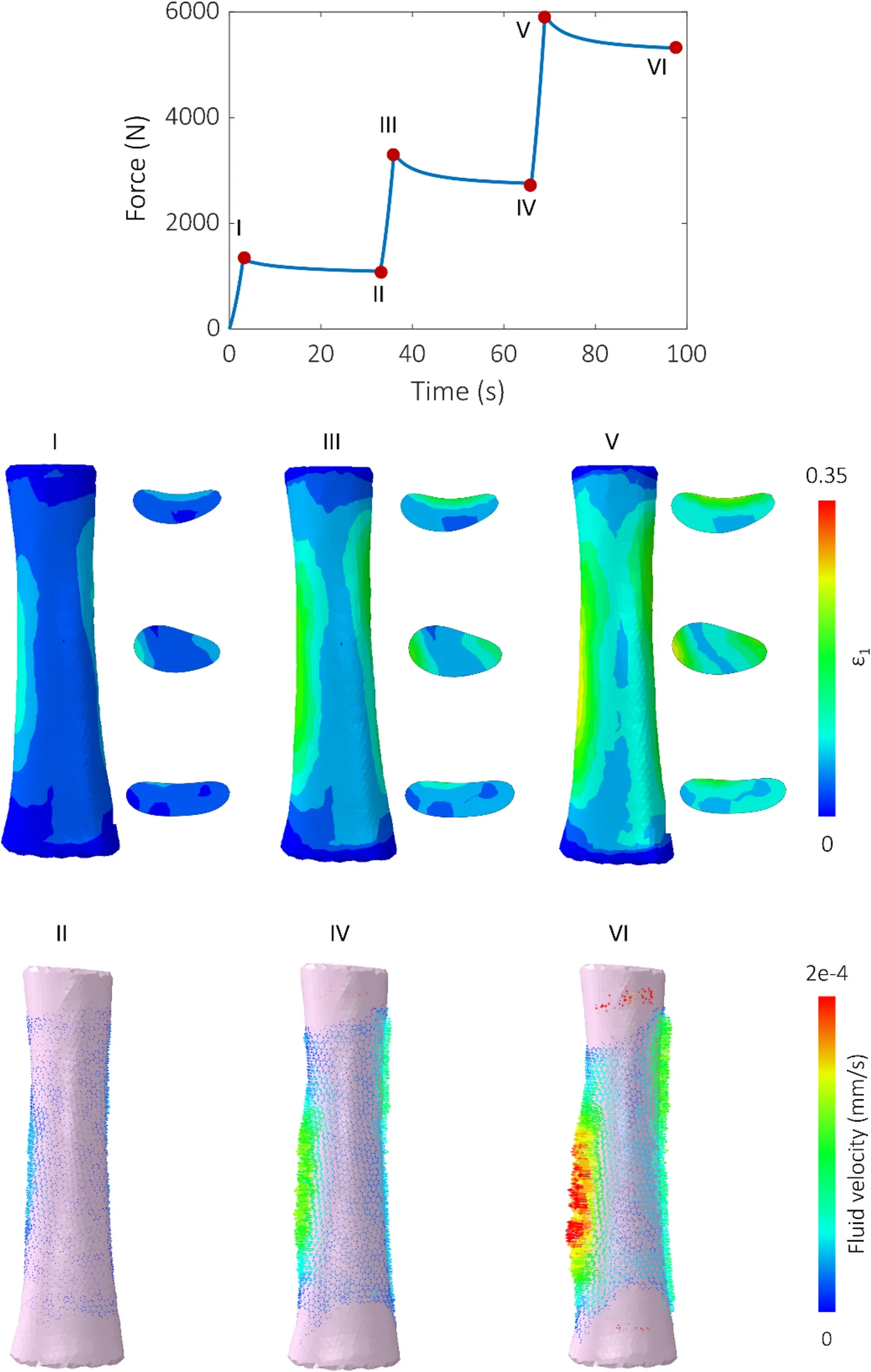
Overall model behavior. The maximum principal strains (*ε*_1_) at the end of the three load steps: 3% (I), 6% (III) and 9% (V) strains are reported on the top row (beside each model, the cross-sectional views at the proximal, the middle and the distal regions are provided) The fluid velocity vectors at the end of the three relaxation steps: 3% (II), 6% (IV) and 9% (VI) are reported on the bottom row

The poro-visco-hyperelastic fiber-reinforced model was able to capture the experimental data well (Fig. 2, Table 2, RMSE < 4%). The model correctly simulated the increased relaxation in the tendon at higher strain levels, as observed experimentally. As no optimization data points were placed in the first peak (3% strain followed by relaxation), a higher discrepancy was observed at the first peak, which was overestimated by the FE model by approximately 20%. Overall, the model exhibited the expected viscoelastic behavior (Fig. 3).

**Table 2:** Collagen material model parameters. Optimized collagen fiber parameters in the SLS model, where *E*_1_, *k*_1_, *E*_2_, *k*_2_ are the stiffness parameters for the non-linear springs and *η* is the damping constant for the dashpot

Optimized parameters				
*E*_1_ (Mpa)	*E*_2_ (Mpa)	*k*_1_	*k*_2_	*η* (Mpa s)
3.10 E+01	0.50 E+00	1.60 E+01	0.66 E+02	0.66 E+02

### Whole tendon model

**Fig. 4:**
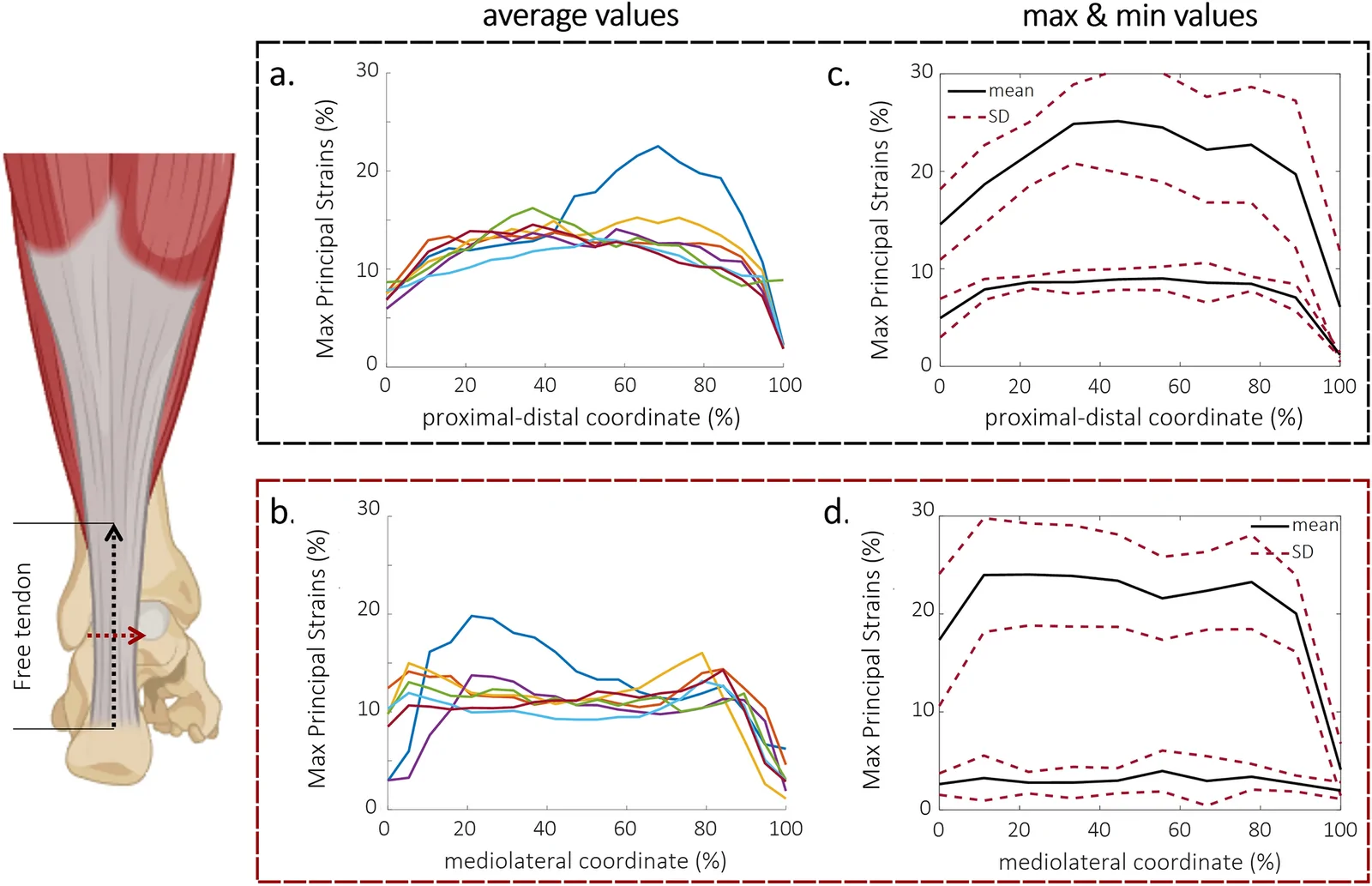
Strain distribution. Max principal strain distribution along the proximal–distal (black) and mediolateral (red) directions. **a**, **b** The mean values for each subject in different colors, and **c**, **d** the minimum and maximum values averaged per all the subjects. The image on the left was generated with help from BioRender

In the whole tendon model, the average strain distribution was consistent across all seven subjects analyzed (Fig. S4). In the proximal–distal direction the models showed a clear trend, with lower strains at the two extremities (the calcaneus insertion and muscle origins) and a region of higher strain in the central portion of the tendon. However, the location of the highest strain varied between 30 and 80% of the free tendon length. The magnitude of the peak strain also varied between subjects, with a between subject variation SD = 20% (Fig. 4a). In the mediolateral direction, the strains were higher closer to the tendon most medial and most lateral locations. Minor peaks were observed at around 20% and 80% of the tendon width, with a plateau in the central portion (Fig. 4b). The between subject variation in the average values was 2.5 times smaller than the between subject variation in the maximum values (Fig. 4c, d).

### The impact of explicitly modeling the subtendons

**Fig. 5:**
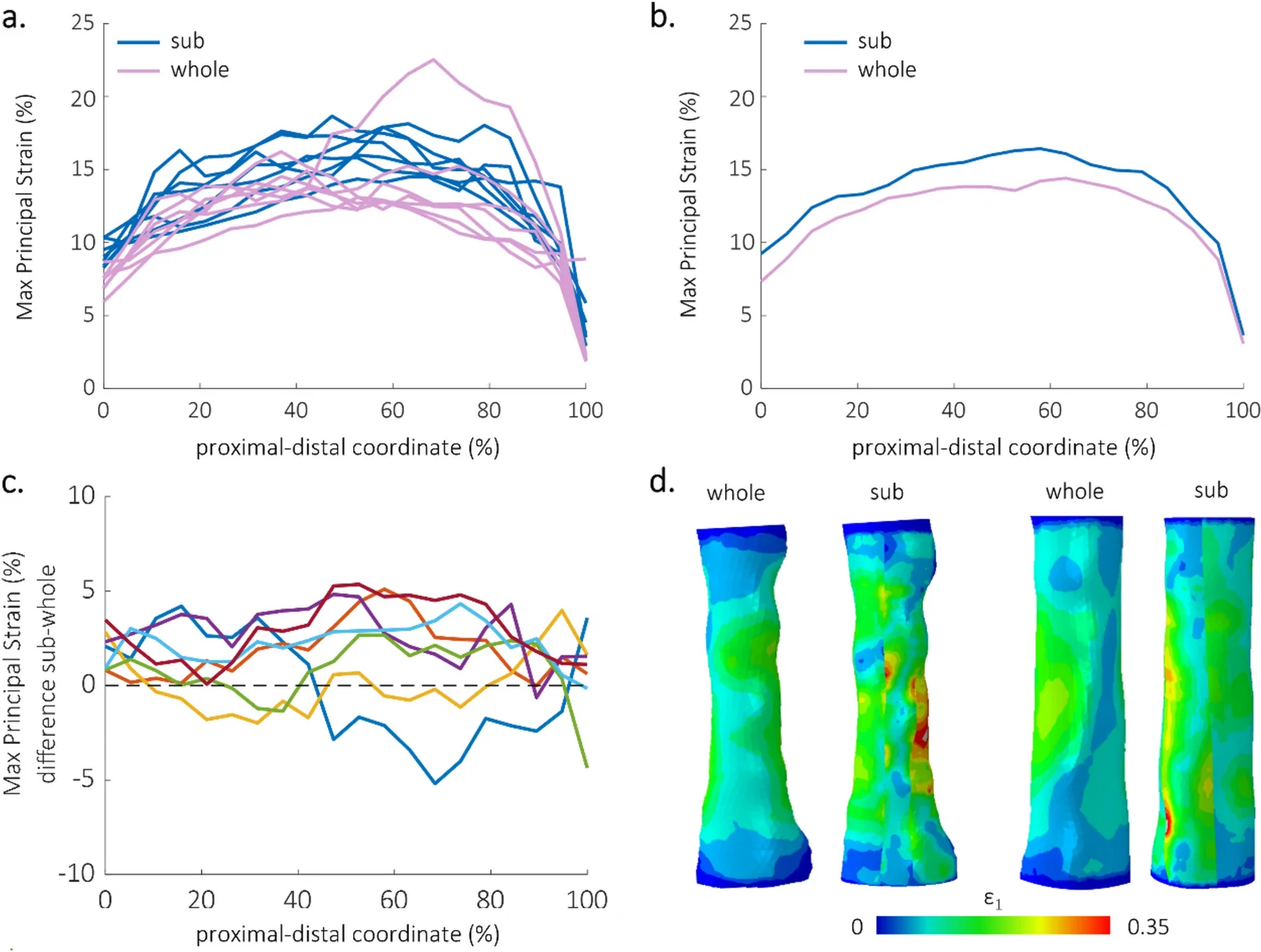
Whole tendon model vs subtendons model. Max principal strain distribution of whole tendon model (pink) and subtendon model (blue) with twisted fibers in the proximal–distal directions are reported **a** separately for each subject analyzed and **b** as the averaged values between all the subjects. **c** The difference in the max principal strains predicted by the subtendon model and the whole tendon model is presented with a different color for each subject analyzed. **d** The contour plots of the maximum principal strains are compared in the posterior view for two representative subjects

In the subtendon models, the average strain distribution was consistent across all seven subjects (Fig. S5). The modeling of the subtendons had no impact on the reaction forces recorded (difference less than 0.1%, Fig. S6). The local heterogeneity of strain values within the tendon was higher in the models that included subtendons (average FWHM of all tendons increased from 0.03 to 0.08). In fact, the subtendon models reached slightly higher strains for all the subjects compared to the whole tendon models (Fig. 5a, b), with only one exception (AT1, see Fig. S4 and S5, and blue line in Fig. 5c).

**Fig. 6:**
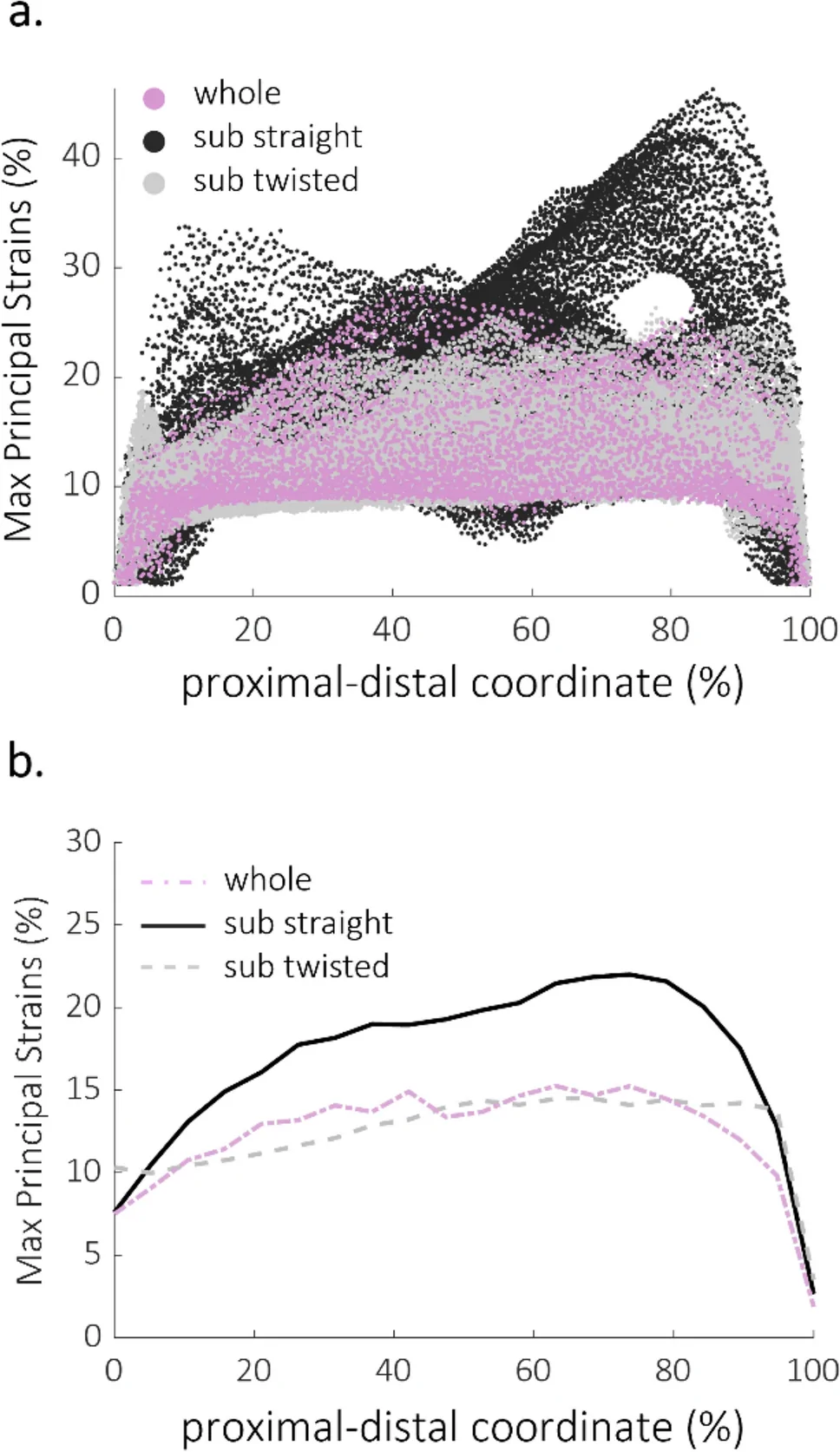
Impact of subtendon modelling on the predicted strains. The values of the principal strains at each node (**a**) and their mean values (**b**) along the tendon length are shown, for the three modelling pipelines tested: whole tendon model (pink), subtendon model with straight fibers (black), subtendon model with twisted fibers (grey)

Changing the main direction of the collagen fibers (following the twisting of the subtendons) strongly impact the strain distribution within the tendon. The FWHM increased from 0.08 for twisted fibers to 0.11 for straight fibers (Fig. 6a). Looking at the mean values of the strains along the proximal–distal direction, the whole tendon model with straight fibers and the model with subtendons with twisted fibers have consistent patterns. In contrast, the strains from the model with subtendons with straight fibers were 8% greater than the other two models (average difference between black line and both pink and gray lines in Fig. 6b).

**Fig. 7:**
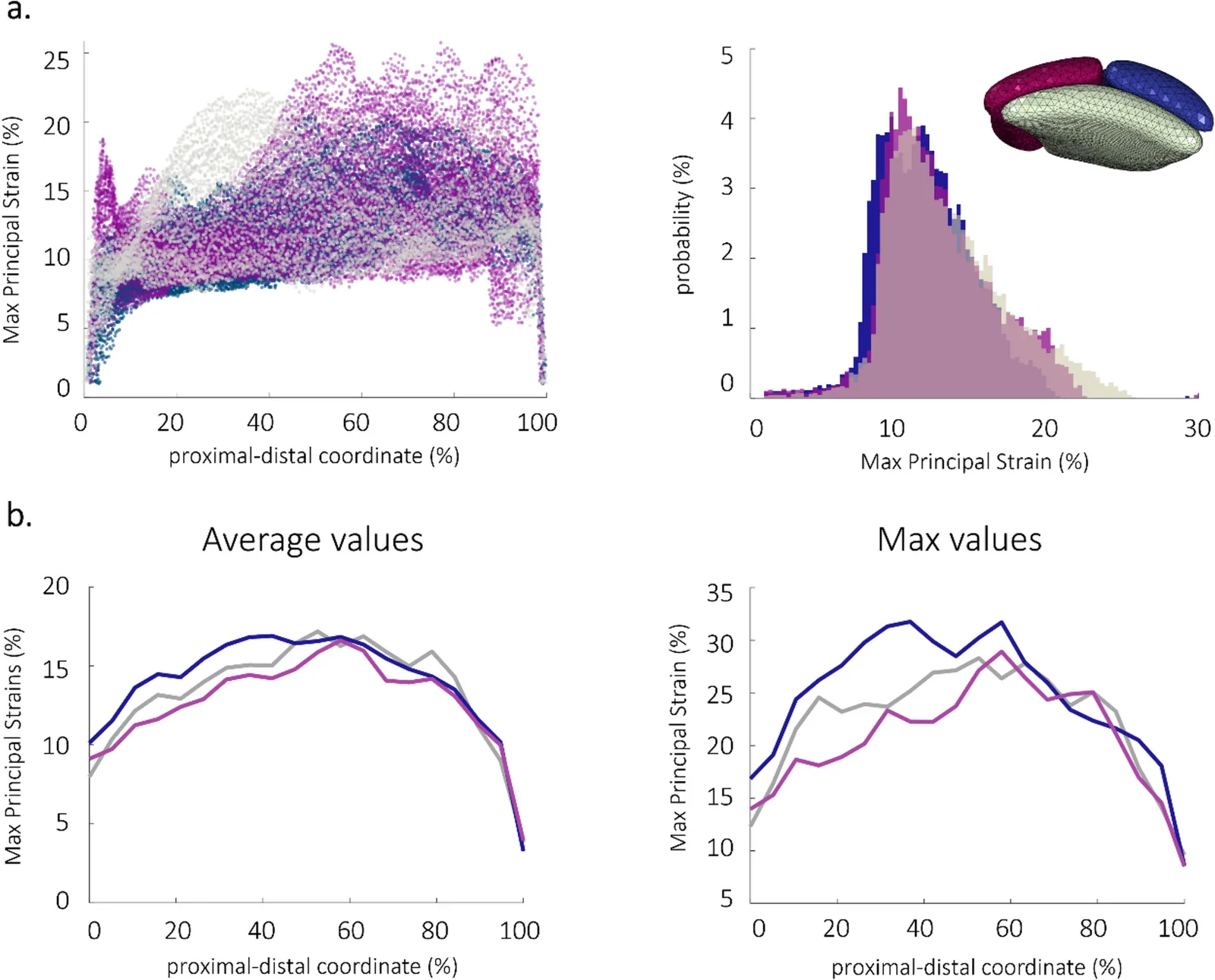
Strain distribution in the three subtendons. The maximum principal strain values at each node of the AT along proximal–distal direction are reported for one representative subject (**a**). The average values (left) and the max values (right) of the maximum principal strains along the subtendon length, i.e. the proximal–distal direction are reported as the mean between the seven subjects (**b**). The results of the three subtendons are reported in the figure using different colors according to the subtendon to which they belong: soleus (white), medial gastrocnemius (blue), and lateral gastrocnemius (violet)

Additionally, the strain distribution inside each one of the three subtendons was analyzed separately. This enabled the identification of the subtendon which exhibited highest localized strains (Fig. 7a). Interestingly, there was no apparent predominance among the three subtendons in either peak strain magnitude or peak strain location. Both the mean and the maximum strain values showed the same trends for the soleus, lateral and medial gastrocnemius subtendon (Fig. 7b).

**Fig. 8:**
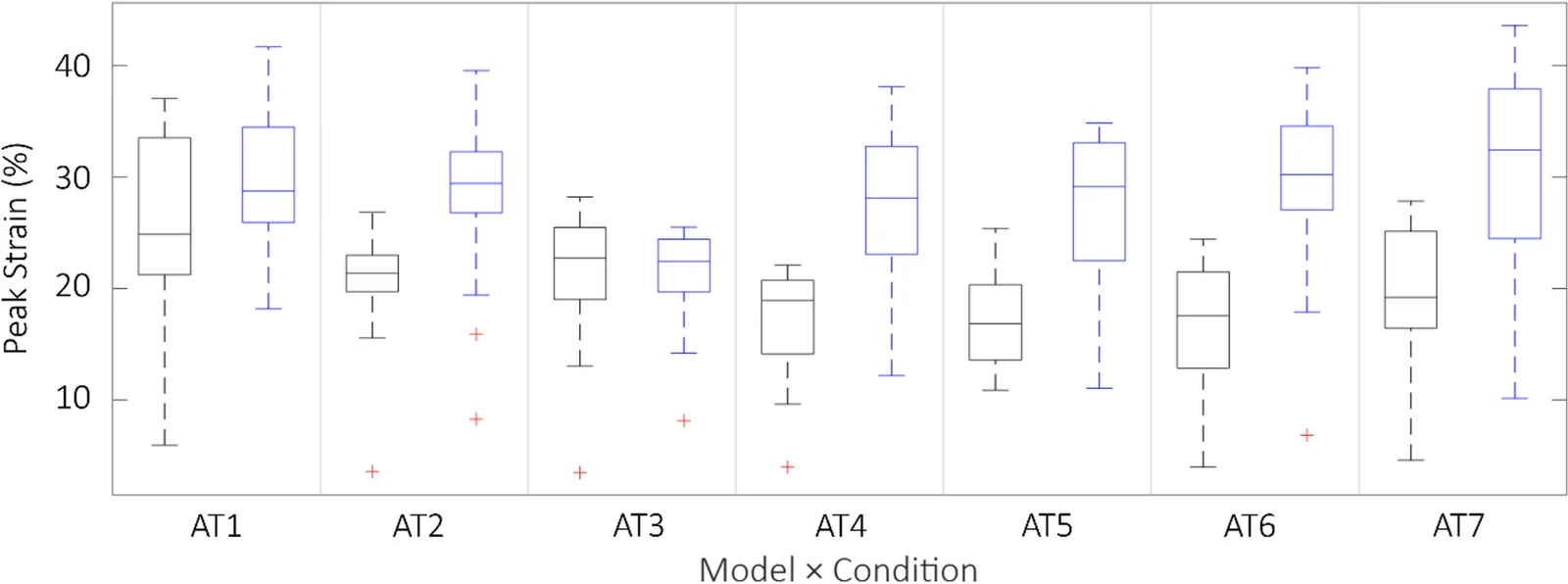
Aligned Rank Transform ANOVA results. Peak strain distribution (%) across seven tendon geometries (AT1–AT7) for whole tendon (black) and subtendon (blue) models. Boxplots show median, interquartile range and outliers for each geometry-condition combination. ART-ANOVA revealed significant effects of tendon geometry, tendon model type and their interaction. Subtendon models generally exhibited higher peak strains than whole tendon models, although the magnitude of this effect varied among geometries

The Aligned Rank Transform ANOVA revealed significant main effects of tendon geometry (*F* = 4.26, *p* < 0.001) and tendon model type (whole tendon vs. subtendon; *F* = 124.15, *p* < 0.001) on peak strain values, as well as a significant interaction between the two factors (*F* = 7.09, *p* < 0.001). Partial eta-squared values on peak strain values indicated a moderate effect of tendon geometry (*η*^2^ₚ = 0.0877), a large effect of tendon model type (*η*^2^ₚ = 0.3182), and a moderate interaction effect (*η*^2^ₚ = 0.1379). Overall, subtendon models exhibited consistently higher peak strain values compared to whole tendon models, although the magnitude of this increase varied between subjects (Fig. 8).

**Fig. 9:**
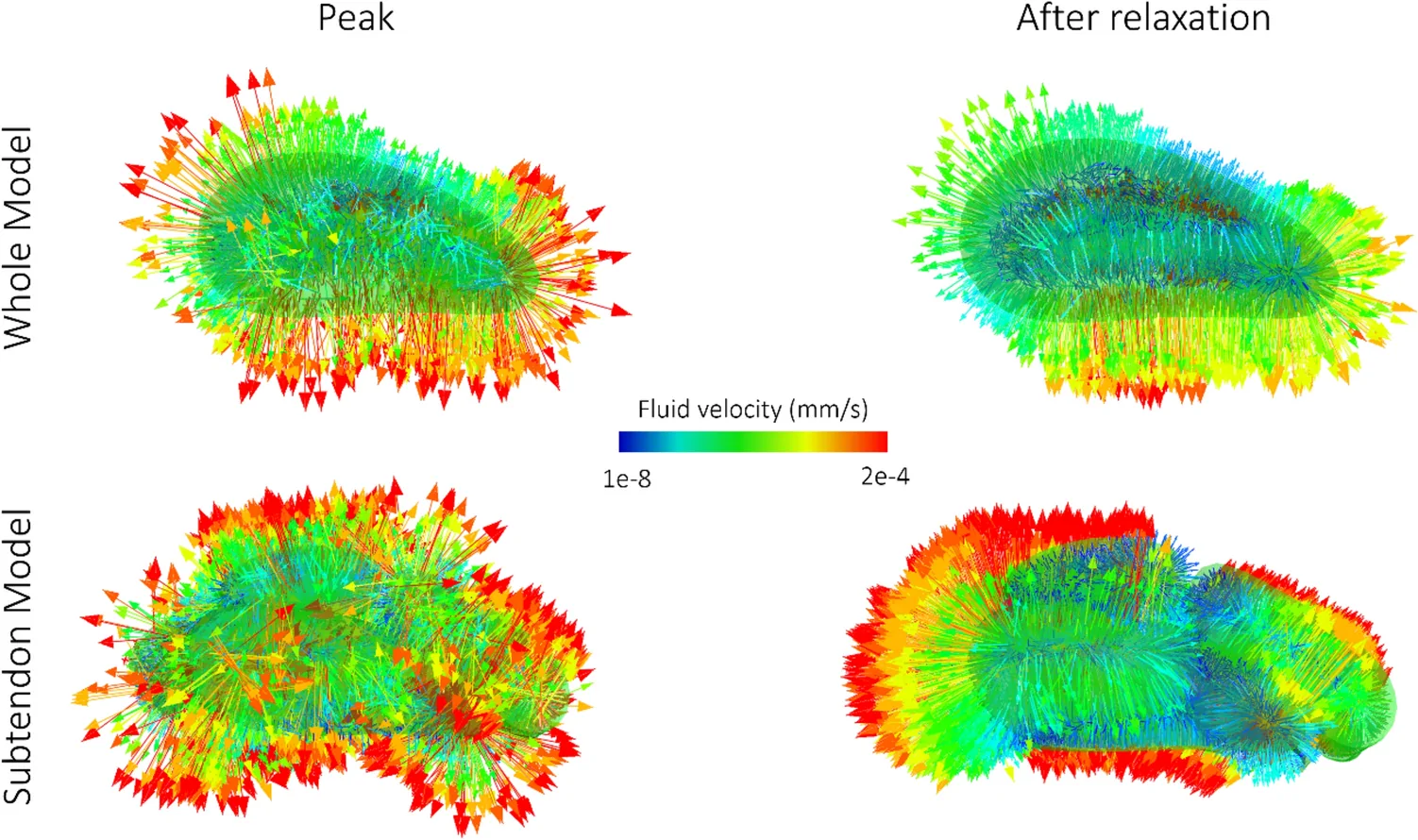
Fluid behavior. The fluid velocity vector is shown for the whole tendon model (top) and the subtendon model (bottom) at 9% strain, at peak load (left) and after relaxation (right). A cross-sectional cut is presented at 25% of the proximal–distal tendon length

The fluid behavior in the whole tendon model and in the subtendon model showed disorganized flow direction at the peak load, Fig. 9. During relaxation, this became regularized, reaching a more organized distribution of the fluid outflow directions at the end of the relaxation. The fluid velocity showed higher values for the subtendon model compared to the whole tendon model, especially at the end of the relaxation step.

## Discussion

In this work, computational models derived directly from clinical MRI were used to investigate the mechanical response of the AT. We focused on the impact of explicitly modeling subtendons on peak strain values, their spatial distribution, heterogeneity and fluid flow and redistribution within the tendon.

The model was able to reproduce the experimentally observed viscoelastic behavior of the tendon tissue (RMSE% < 4%). Some discrepancies were noticed at the first load step (3% strain), which the model overestimated. This could be related to relaxation or some slacks at the clamping site experimentally. For this reason, our analysis was primarily focused on the model behavior at higher applied loads (9% strain).

The findings highlighted that average strains showed similar trend across all participants, but the magnitude and the localization of the peak strains varied. Specifically, the magnitude varied by 20%, with its location occurring somewhere between 30 and 80% of the tendon free length. As the same material properties were used for all tendons, this variation is attributed solely to subject-specific geometry. These findings are consistent with the results from Hansen et al. (2017), which indicate that the stress distribution in a healthy, free AT during loading is more influenced by individual tendon geometry than by subject-specific material properties. However, in their study, the same volume mesh was morphed to patient ultrasound images, whereas in the present study, each model was generated directly from each patient´s MRI data. Additionally, clinical literature reports that the AT midsubstance is the region with higher risk of failure (Szaro et al. 2021), but the specific location varies between subjects, within a range of 2–6 cm from the calcaneal insertion (Gwynne-Jones and Sims 2011), and our findings are within that range. Both the modeling approaches, and more prominently the subtendon approach, predicted localized strains up to three times higher than the nominal strain. This finding is consistent with both computational and experimental literature. Indeed, Funaro et al. (Funaro et al. 2022) reported local strains during rehabilitation exercises that were nearly one order of magnitude greater than the average strain values. Similarly, using DIC techniques during mechanical testing, Nagelli et al. (Nagelli et al. 2022) experimentally observed localized strains approximately twice as high as the applied average tendon strain. Furthermore, the comparison of the three model pipelines (the whole tendon model, the subtendon model without twisted fibers and the subtendon model with twisted fibers) revealed that while the average strains in models that explicitly include the subtendons were comparable to those in models that did not, local heterogeneity was significantly greater in the subtendon models. Additionally, the average strains in the subtendon models with twisted fibers were more closely aligned with those of the whole tendon model than those observed in subtendons with straight fibers. Therefore, the explicit modeling of subtendons, as well as the orientation of the fibers, plays a crucial role in the distribution of strain.

Our results show that accounting for the subdivision of the three subtendons is important, not only to enable replicating rehabilitation tasks (Funaro et al. 2022) but also because analyzing each component separately pointed to that the high-strain location along the tendon length is personalized. Previous studies have raised this aspect qualitatively (Handsfield et al. 2020; Knaus and Blemker 2021; Yin et al. 2021). In this study, we confirm quantitatively an increase in the FWHM from 0.03 to 0.08 from the whole tendon model to the subtendons model.

Additionally, using a poro-visco-hyperelastic model enabled the fluid flow in the tendon to be captured. Fluid flow is known to enhance tendon viscoelasticity (Yin and Elliott 2004). It was found that accounting for fluid flow resulted in a different redistribution of the internal strains compared to a purely viscoelastic material description (Fig. S7). This poro-visco-hyperelastic material model has earlier been presented and validated for rat AT modeling (Khayyeri et al. 2016; Notermans et al. 2019). Explicit modeling of the fluid flow may also be of relevance for future mechanobiological predictions.

Taken together, these findings are important in the work toward using finite element models to predict tendon tissue regeneration and to explore rehabilitation strategies following injuries, as tendons are known to be mechanoregulated (Wang 2006; Andersson et al. 2009; Hammerman et al. 2018). Our group has previously presented a framework where mechanobiological stimulations based on strain showed the ability to predict tissue regeneration after tendon transection in a rat model (Notermans et al. 2021, 2022; Notermans and Isaksson 2023). As a precursor for translating that work toward human tendon healing, the current study showed that it is critical to incorporate subject-specific tendon shape.

Some limitations need to be underlined. First, the models were generated from MR images which did not allow for a clear personalized distinction between the subtendons. The subtendons division was thus idealized, following the procedure earlier used by Knaus and Blemker (2021). Average twisting based on the literature was used; however, this may not be correct for each subject. As newer peripheral higher-resolution MRI is introduced into clinical practice (Szaro et al. 2020), we will evaluate the importance of subject-specific segmentation of the subtendons in subsequent studies. In the current study our focus was instead on maximizing the information extracted from established MRI sequences and evaluating the importance of the subtendon division and fiber orientation on strain distribution. Secondly, even if the material model exploited has been per se previously validated, a validation of the human model outcomes in term of strain distribution is still missing. This is something we are currently addressing in a separate study. Thirdly, the material parameters were determined by fitting the experimental data to one of the tendons and then using the identified parameter set (Table 2) for all the seven tendon samples. Optimizing the properties for each tendon may have resulted in a slightly different material parameter set for each tendon, but most likely it would not affect the location of maximum strains or the level of heterogeneity within the tendon. Lastly, in the subtendon model with twisted fibers, all fibers were aligned in the direction of the subtendons. This may simplify strain distributions in areas with a higher risk of failure. Therefore, it would be beneficial to compare this idealized method of fiber orientation with the actual local fiber distribution. This is not trivial, but has recently been shown possible based on synchrotron phase contrast X-ray tomography of small animal tendons (Pierantoni et al. 2023; Janssen et al. 2025). Tendon tissue can also be analyzed at sub-micrometric level by combining focused ion beam milling with scanning electron microscopy, but this is destructive technique and has a limited field of view (less than 100 × 100 µm) (Svensson et al. 2017). Alternatively, confocal or multiphoton microscopy can be employed, however, it is important to note that this technique is limited to samples with a maximum thickness of 500 µm due to its restricted penetration depth (Theodossiou et al. 2006). Our group recently explored the use of data from synchrotron phase contrast X-ray tomography for simulating the mechanical response of rat AT (Janssen et al. 2025), showing that including local fiber distribution in the tendon material model has a significant impact on the strain distributions. This study simulated the behavior of fully loaded and unloaded rat AT and demonstrated that the altered mechanical response after unloading is mainly due to altered fiber distribution. However, until now, the acquisition of phase contrast X-ray tomography has mainly been possible for smaller samples (mm-sized). Nevertheless, the recent development of the BM18 beamline at ESRF and The Human Organ Atlas Hub (Walsh 2021) opens the possibility of acquiring high resolution images of whole human tendons.

In conclusion, this study quantitatively analyzed the impact of explicit subtendon modeling and the orientation of the main fiber direction in the generation of AT finite element models. The results indicated that subtendons should be treated as separate entities in the modeling process and that realistic fiber orientation should be taken into account, as both these aspects significantly affect the distribution and heterogeneity of strain within the tendon structure. Future research will build on this foundation by incorporating subject-specific fiber orientations into the modeling pipeline.

## Supplementary Information

Below is the link to the electronic supplementary material.

Supplementary file1 (PDF 818 kb)

## Data Availability

Data are provided within the manuscript or supplementary information files.
