# Supplementary material for: Finite element modeling of human Achilles tendon: impact of subtendon modeling and fiber direction
Source: Biomech Model Mechanobiol. 2026 Sep 7;25(5):99. doi: 10.1007/s10237-026-02132-z (PMC13550095; doi:10.1007/s10237-026-02132-z)
Supplement: Supplementary file 1 [file 10237_2026_2132_MOESM1_ESM.pdf]

SUPPLEMENTARY MATERIALS

FINITE ELEMENT MODELLING OF HUMAN ACHILLES TENDON: IMPACT OF SUBTENDON MODELLING AND FIBER DIRECTION

Chiara Garavelli (1), Pernilla Eliasson (2,3), Hanna Isaksson (1)

1. Department of Biomedical Engineering, Lund University, Sweden
2. Department of Orthopaedics, Institute of Clinical Sciences, Sahlgrenska Academy, University of Gothenburg, Goteborg, Sweden.
3. Department of Orthopaedics, Sahlgrenska University Hospital, Sweden

DOI: 10.1007/s10237-026-02132-z

Supplementary material S1: Material model description and numerical implementation

Material model

The tendon tissue is modelled using a material model that largely stems from the model originally presented by Wilson et al. (Wilson et al. 2005) for articular cartilage. The model has been used extensively for many different soft tissues, and we previously extended this formulation to a poro-visco-hyperelastic material able to capture the mechanical behavior of tendon (Khayyeri et al. 2015, 2016). Below, we are recapitulating the mechanical behavior of the model,

The total stress is derived from three different components: the stress in the collagen fibers, the stress in the ground substance and the stress from the fluid flow:

$$\boldsymbol{\sigma}_{tot} = \boldsymbol{\sigma}_f + \boldsymbol{\sigma}_m - p\mathbf{I}$$

Where $\boldsymbol{\sigma}_{tot}$ is the total stress, $\boldsymbol{\sigma}_f$ is the stress in the collagen fibers, $\boldsymbol{\sigma}_m$ is the stress in the non-fibrillar matrix and p is the hydrostatic pressure with unit tenson $\mathbf{I}$ from the fluid.

The contributions of the three components are explained below.

1) *Collagen*

For the fibrillar component a standard linear solid model is used (see main manuscript Fig.1e) with a spring in parallel to a series of a spring and a damper to capture the viscoelastic features. The springs are considered to have an exponential stress-strain relationship, and collagen is assumed to only bear load in tension:

$$\boldsymbol{\sigma}_1 = \begin{cases} E_1\left(e^{k_1\varepsilon_f} - 1\right) & \varepsilon_f > 0 \\ 0 & \varepsilon_f \leq 0 \end{cases}$$

$$\boldsymbol{\sigma}_2 = \begin{cases} E_2\left(e^{k_2\varepsilon_e} - 1\right) & \varepsilon_f > 0 \\ 0 & \varepsilon_f \leq 0 \end{cases}$$

$$\boldsymbol{\sigma}_2 = \eta\,\dot{\varepsilon}_d$$

The total fiber stress is: $\boldsymbol{\sigma}_f = \boldsymbol{\sigma}_1 + \boldsymbol{\sigma}_2$

2) Non-fibrillar matrix

The constitutive response of the non-collagenous matrix is described by a finite-strain transversely isotropic elastic constitutive law:

$$\boldsymbol{W}_{orthogonal} = \frac{1}{2}\sum_{i,j}^{3} a_{ij}tr(E \cdot L_{ii})tr(E \cdot L_{jj}) + \sum_{i,j \neq 1}^{3} G_{ij}tr(E \cdot L_{ii} \cdot E \cdot L_{jj})$$

Where E is the Green-Lagrange strain tensor and $L_{ii} = l_i \otimes l_i$ (i = 1,2,3) is a set of orthogonal unit base vectors with transversal (i = 1,3) and longitudinal (i = 2) orientation, and a_{ij} (i,j = 1,2,3) is the stiffness tensor, which components are:

$$a_{ii} = E_i \frac{1-\nu_{jk}\nu_{kj}}{1-\nu_{12}\nu_{21}-\nu_{23}\nu_{32}-\nu_{31}\nu_{13}-2\nu_{21}\nu_{32}\nu_{13}},$$

$$a_{ij} = E_i \frac{\nu_{ij} + \nu_{jk}\nu_{ik}}{1-\nu_{12}\nu_{21}-\nu_{23}\nu_{32}-\nu_{31}\nu_{13}-2\nu_{21}\nu_{32}\nu_{13}},$$

$$i \neq j \neq k$$

Consequently, the transversely isotropic stiffness tensor is fully characterized by five independent material parameters: the longitudinal Young's modulus E_n, the transverse Young's modulus E_p, the longitudinal-transverse Poisson's ratio ν_{pn}, the transverse Poisson's ratio ν_p, and the longitudinal shear modulus G_{pn}.

$$E_1 = E_3 = E_p, \qquad E_2 = E_n,$$
$$\nu_{13} = \nu_p, \nu_{12} = \nu_{32} = \nu_{pn},$$
$$G_{13} = \frac{E_p}{2(1+\nu_p)},$$
$$G_{12} = G_{23} = G_{pn}$$

The Cauchy stress in the non-fibrillar matrix is given by:

$$\boldsymbol{\sigma}_m = \frac{1}{J}\mathbf{F} \cdot \frac{\partial W}{\partial E} \cdot \boldsymbol{F}^{\boldsymbol{T}}$$

$$\boldsymbol{\sigma}_m = \frac{1}{J}\mathbf{F} \cdot \left[\sum_{i,j}^{3} a_{ij}tr(E \cdot L_{jj})\, L_{ii} + 2\sum_{i,j \neq 1}^{3} G_{ij}L_{ii} \cdot E \cdot L_{jj}\right] \cdot \boldsymbol{F}^{\boldsymbol{T}}$$

3) Fluid

The fluid flow is inversely proportional to the permeability k and the pore pressure gradient ∇p, following Darcy law. The permeability, k, is assumed to be strain-dependent and is given by

$$k = k_0\left(\frac{1+e}{1+e_0}\right)^{M_k}$$

where k_0 is the initial permeability, M_k is a positive constant and e and e_0 are the current and initial void ratios. The void ratio in a porous medium is defined as the volume ratio between fluid and solid

$$e = \frac{n_f}{n_s}$$

The total water volume fraction, n_f, was estimated from the water mass fraction $n_{f,m}$ (Wilson et al. 2007) as

$$n_f = \frac{\rho_s n_{f,m}}{1 - n_{f,m} + n_{f,m}\, \rho_s}$$

where ρ_s is the assumed solid tissue density.

Numerical implementation of the material model

A poroelastic approach is used, where a subroutine for user-defined materials (Abaqus UMAT) is used to define the material model of the solid matrix (ground substance and collagen fibers). To account for the large deformations an updated Lagrangian formulation was used.

The fibers are represented by direction vectors, where e^n corresponds to the reference configuration and e^{n+1} the deformed configuration. The fiber stretch is defined as the length ratio between the direction vectors

$$\lambda = \frac{|e^{n+1}|}{|e^n|}$$

The strain increment is calculated as the logarithmic strain from the stretch

$$\Delta\varepsilon = ln\left(\frac{|e^{n+1}|}{|e^n|}\right) = \ln(\lambda)$$

and the total strain in increment *n+1* was: $\varepsilon^{n+1} = \varepsilon^n + \Delta\varepsilon$

In each iteration the fiber direction vectors are updated, and the deformed direction vectors are calculated from the deformation gradient **F** as: $e^{n+1} = \boldsymbol{F}e^n$

References

Khayyeri H, Gustafsson A, Heuijerjans A, et al (2015) A Fibre-Reinforced Poroviscoelastic Model Accurately Describes the Biomechanical Behaviour of the Rat Achilles Tendon. PLoS ONE 10:e0126869. https://doi.org/10.1371/journal.pone.0126869

Khayyeri H, Longo G, Gustafsson A, Isaksson H (2016) Comparison of structural anisotropic soft tissue models for simulating Achilles tendon tensile behaviour. Journal of the Mechanical Behavior of Biomedical Materials 61:431–443. https://doi.org/10.1016/j.jmbbm.2016.04.007

Wilson W, Huyghe JM, Van Donkelaar CC (2007) Depth-dependent Compressive Equilibrium Properties of Articular Cartilage Explained by its Composition. Biomech Model Mechanobiol 6:43–53. https://doi.org/10.1007/s10237-006-0044-z

Wilson W, Van Donkelaar CC, Van Rietbergen B, Huiskes R (2005) A fibril-reinforced poroviscoelastic swelling model for articular cartilage. Journal of Biomechanics 38:1195–1204. https://doi.org/10.1016/j.jbiomech.2004.07.003

Supplementary material S2: Full-Width Half-Maximum derivation

The Full-width Half-maximum is derived from the strain histogram distributions. The values reported are respectively the average between all the subjects for the whole model and for the subtendon models.

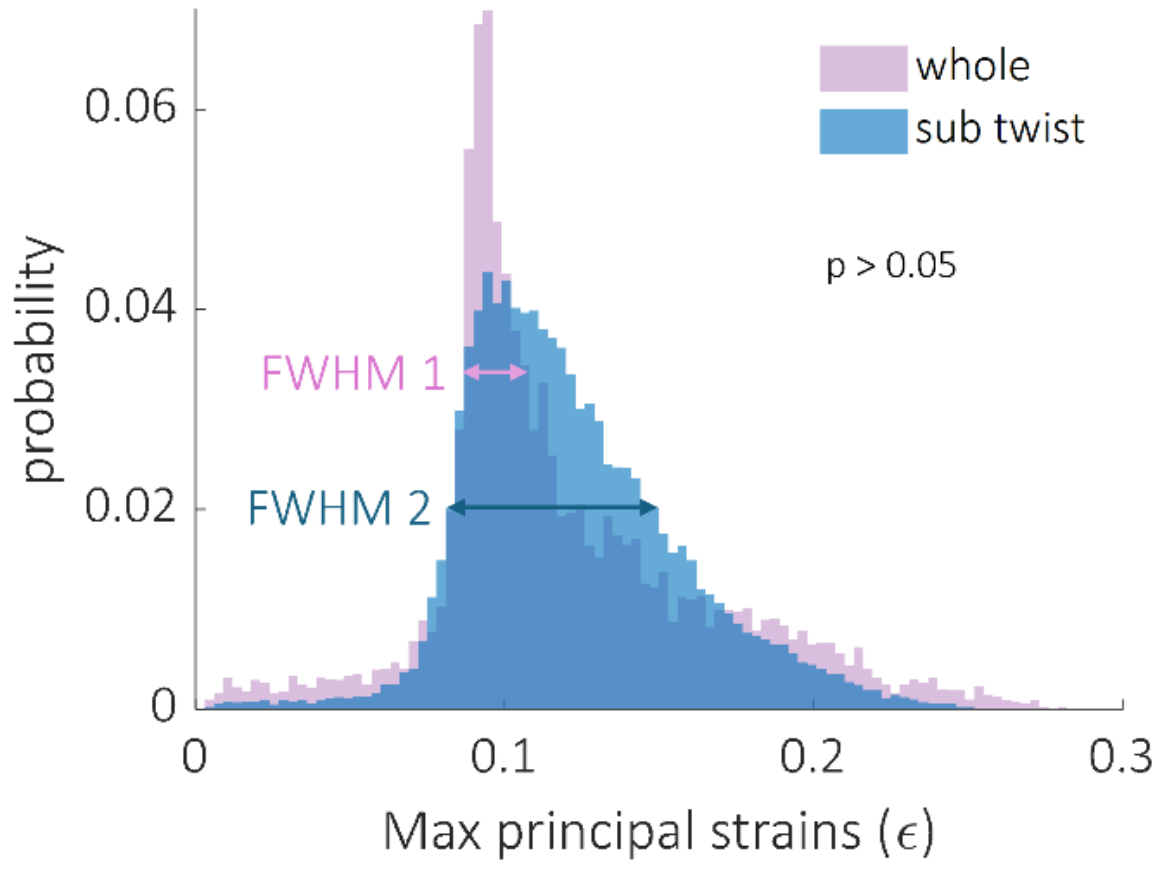

Full-width Half-maximum analysis. For each sample, the Full-width Half-maximum is derived from the strain histogram distributions of the whole approach and the subtendon with twisted fiber approach. In the figure, the example from one sample.

Supplementary figures

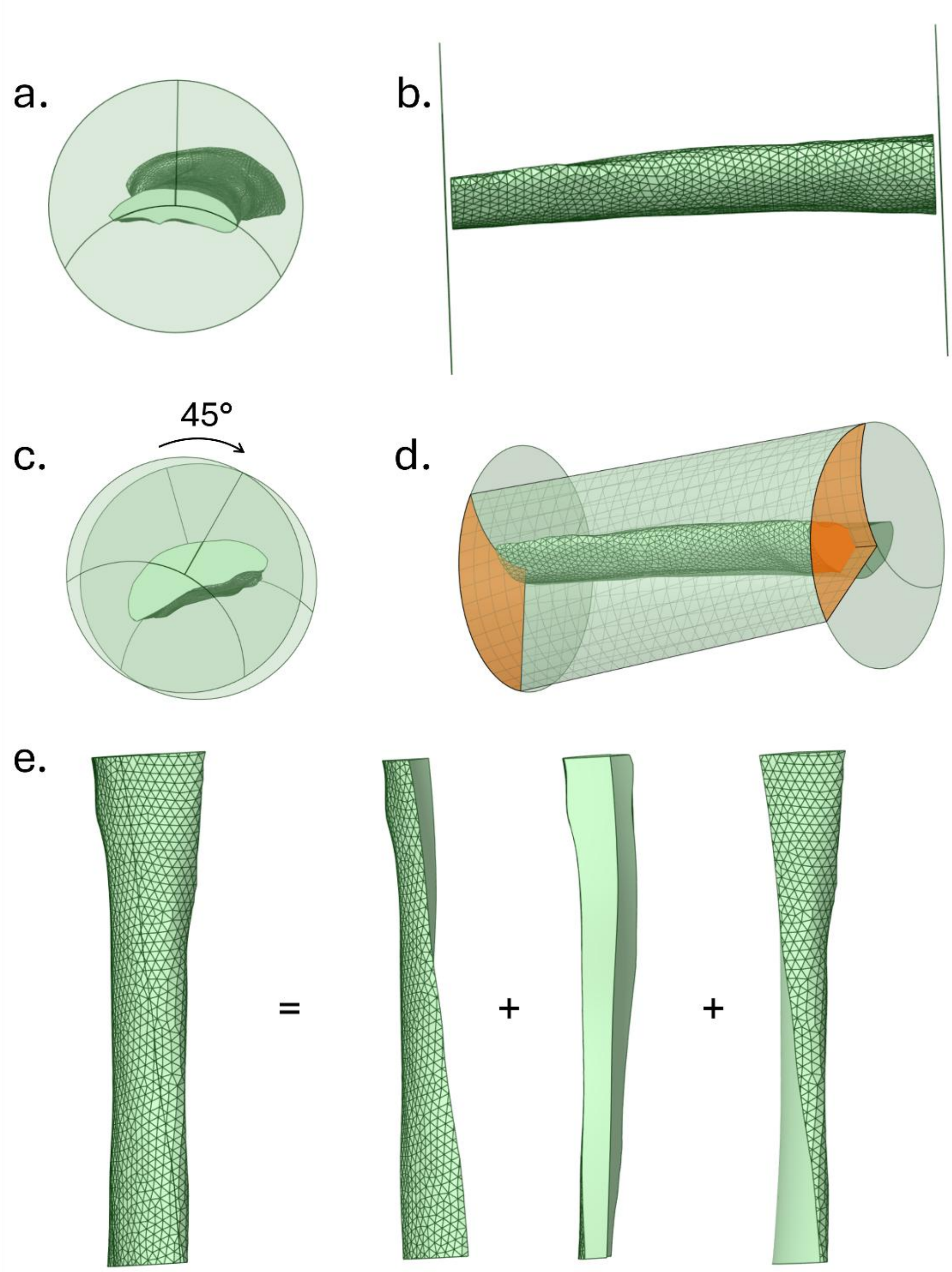

Fig. S2: Steps for the subtendons division. a) A surface mask was created at the proximal extremity of the free tendon. The lower part represents the soleus, since it is always located more internally, while the two upper parts represent the two gastrocnemii. b) The mask was copied and translated at the calcaneus extremities of the model and c) rotated 45 degrees. d) A volume was created connecting each region in the two masks (orange). The lateral surfaces of the generated volume was used as cutting surfaces. e) Posterior view of the tendon with the three subtendons attached all together (left) and then split apart (right).

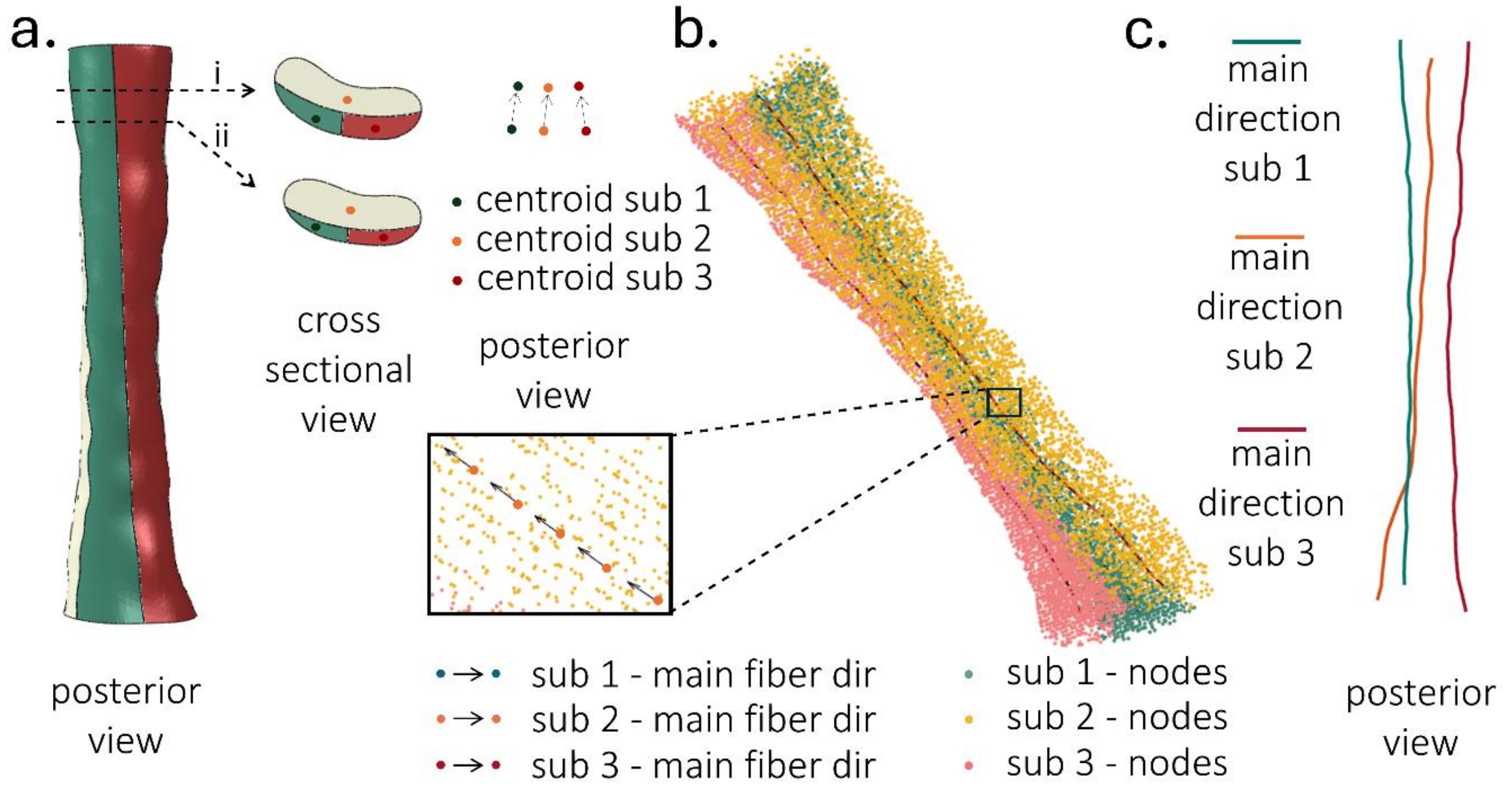

Fig. S3: Approach to assigning the fiber direction for each element. a) The direction of the collagen fiber in each element was defined by first dividing the tendon length into approximately 2-mm subsections, where the centroid of each subsection was computed. b) Then the centroids were connected to each other within one subtendon, and finally c) the fiber direction was defined following the centroids.

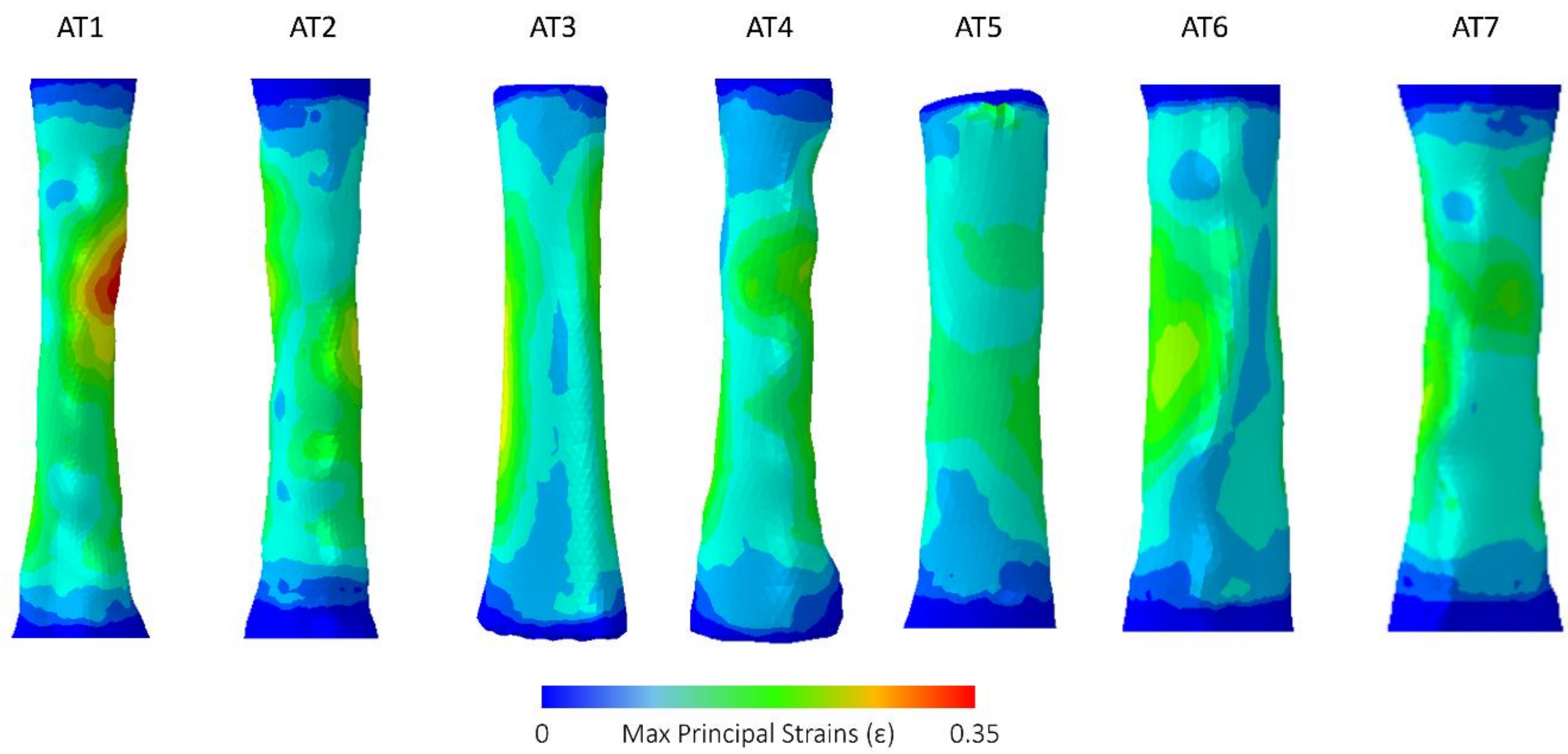

Fig. S4: Strains distribution dependency on the tendon geometry for the whole model approach. The contour plot of the maximum principal strains of the whole tendon model for each one of the tendons simulated at 9% peak strain (posterior view). They clearly show the impact of geometry on strain distribution.

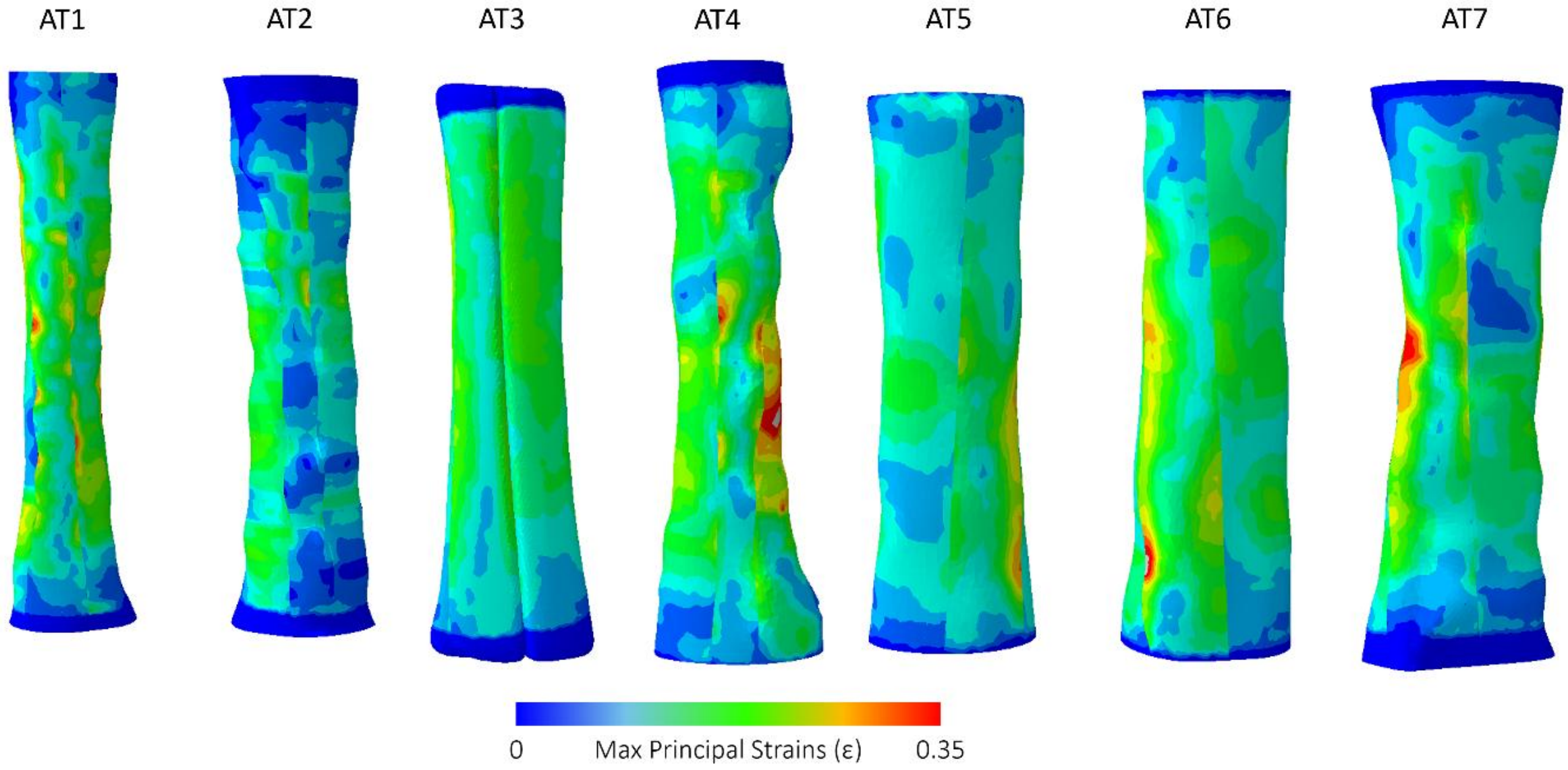

Fig. S5: Strains distribution dependency on the tendon geometry for the sub-tendon model approach. The contour plot of the maximum principal strains of the sub-tendon model for each one of the tendons simulated at 9% peak strain (posterior view).

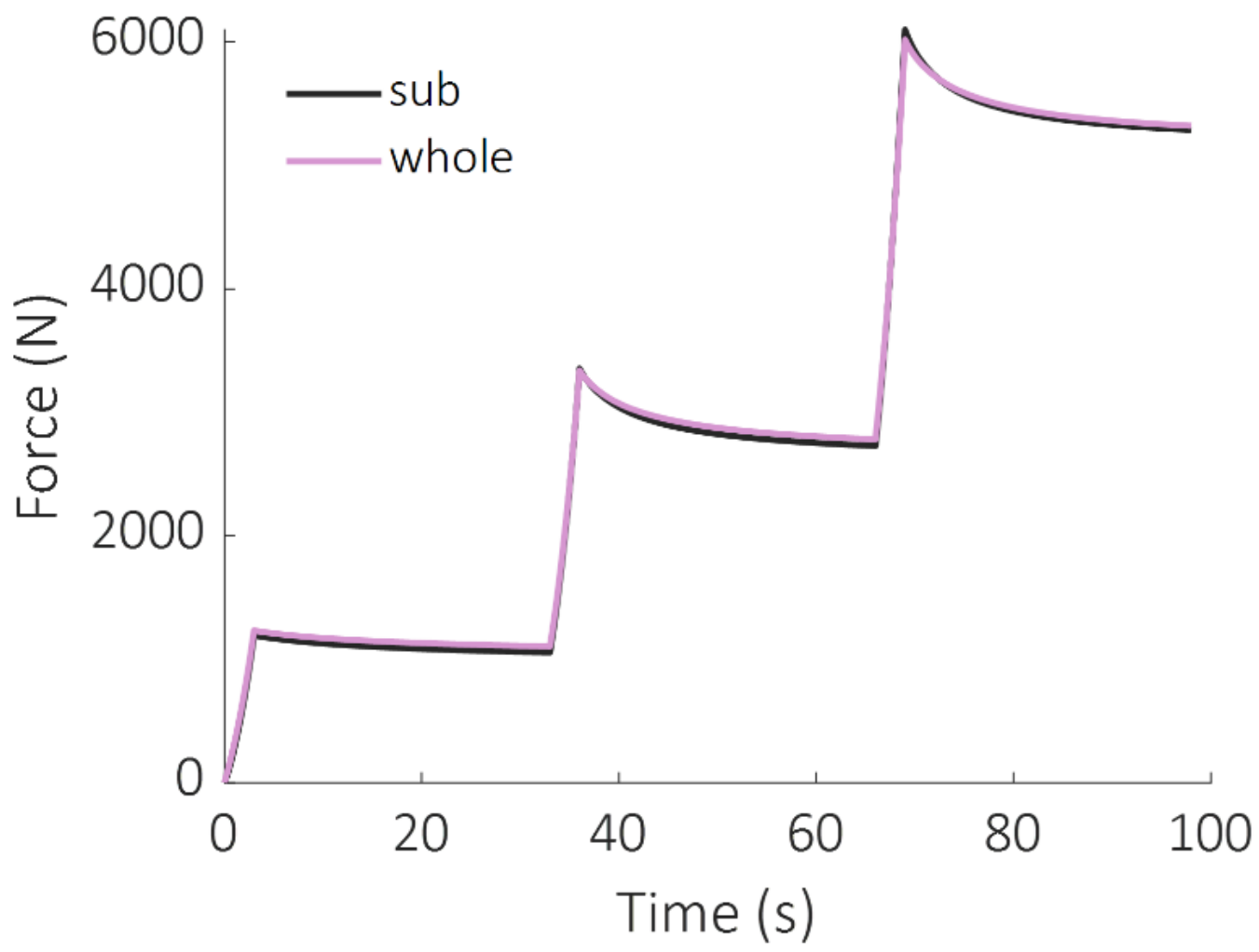

Fig. S6: Force reactions of whole and sub-tendon model. The rection forces predicted by the subtendons model (black) and the whole tendon model (pink) during the whole test are reported in figure. Modeling sub-tendons had no visible impact on the reaction forces predicted by the models, with a difference lower than 0.1%.

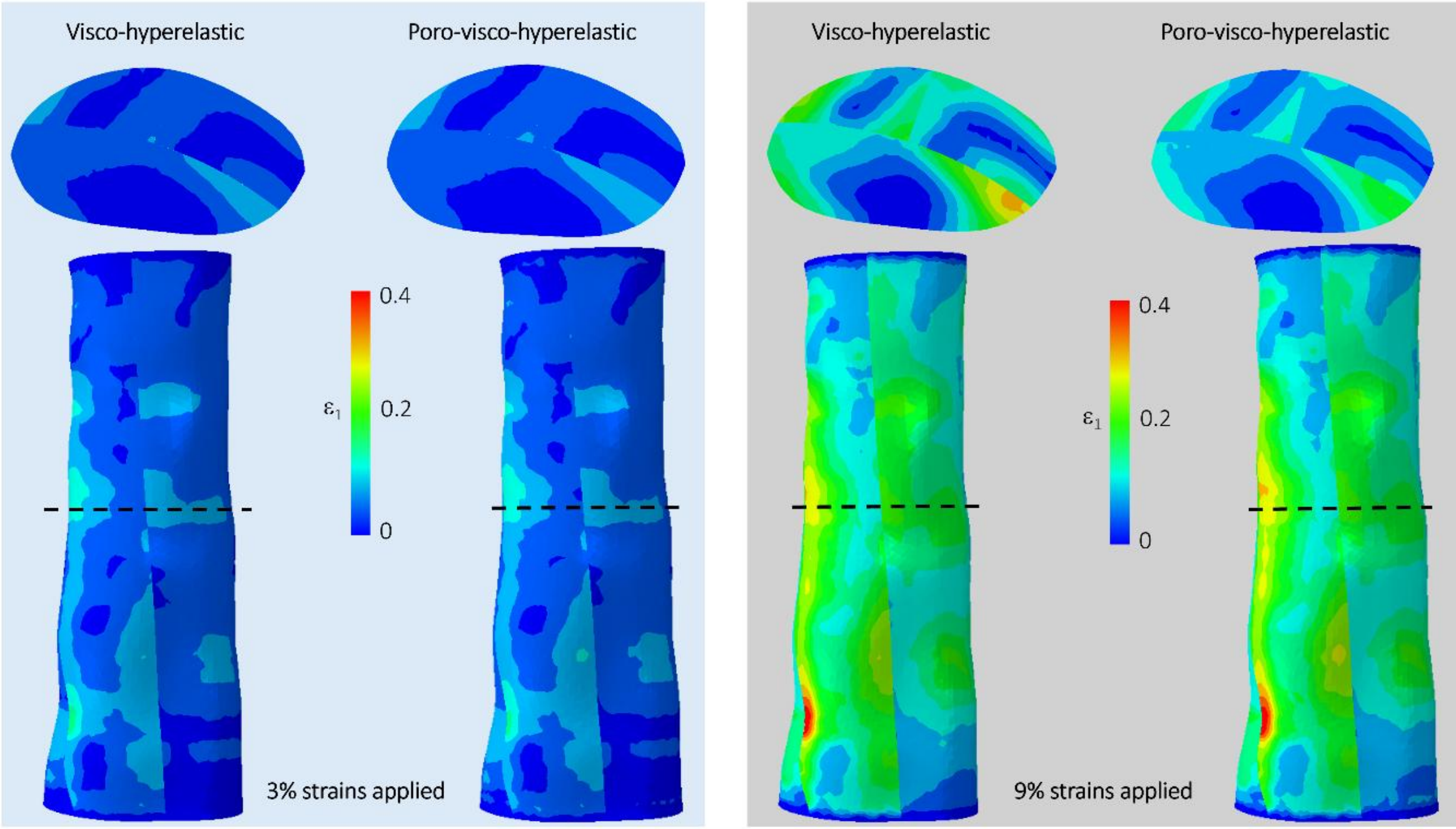

Fig. S7: Impact of the inclusion of the fluid component in the material model on the strain distribution. The same Achilles tendon geometry was tested with the poro-visco-hyperelastic material model used in this work and with a similar material model where the fluid component was removed, without altering other parameters. The strain distribution at the surface (posterior view) and at one section (half of tendon length) were compared after the first and the last pulling step (corresponding to 3% and 9% respectively), after the relaxation period, to assess impact of the inclusion of the fluid component in the modelling.
